# Modulating the cobalt redox potential through imidazole hydrogen bonding interactions in a supramolecular biomimetic protein-cofactor model†
†Electronic supplementary information (ESI) available. See DOI: 10.1039/c5sc04396d


**DOI:** 10.1039/c5sc04396d

**Published:** 2016-02-23

**Authors:** Marjorie Sonnay, Thomas Fox, Olivier Blacque, Felix Zelder

**Affiliations:** a Department of Chemistry, University of Zurich , Winterthurerstr. 190, CH-8057 , Zurich , Switzerland . Email: felix.zelder@chem.uzh.ch

## Abstract

A realistic model for the active site of histidine-on cobalamin@protein complexes is reported and studied under homogeneous and immobilized conditions. Analysis of lower ligand modulation and its influence on the properties of the biomimetic compound are presented. The cofactor attachment by a protein's histidine residue was imitated by covalently linking an artificial imidazole-containing linker to cobyric acid. The resulting intramolecular coordination complex is an excellent structural model of its natural archetype, according to 2D ^1^H-NMR studies and molecular modeling. The effect of deprotonation of the axially coordinating imidazole ligand – as proposed for natural cofactor complexes – tunes significantly the position of the cathodic peak (Δ*V* = –203 mV) and stabilizes thereby the Co^III^ form. Partial deprotonation of the imidazole moiety through hydrogen bonding interactions was then achieved by immobilizing the biomimetic model on hydrophobic C18 silica, which yielded an unprecedented insight on how this class of Cbl-dependent proteins may fine-tune their properties in biological systems.

## Abstract 2

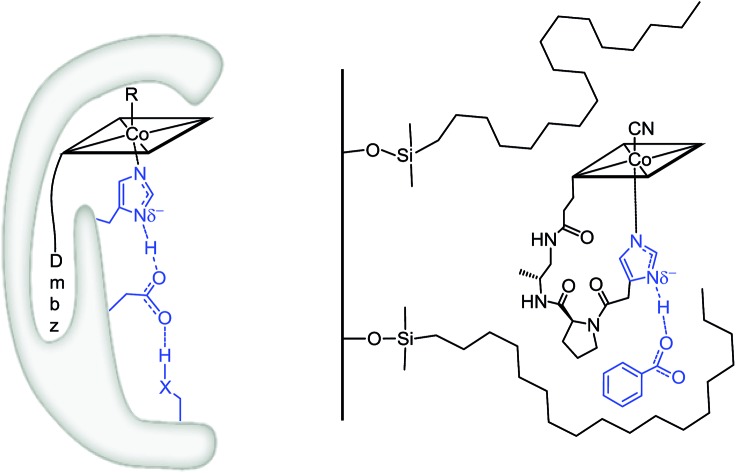
This paper describes a supramolecular biomimetic model of the “His-on” configuration and the charge relay system present in certain types of B_12_-dependent enzymes.

## 

The vitamin B_12_ (“B_12_”) cofactors methylcobalamin (MeCbl) and adenosylcobalamin (AdoCbl) represent organometallic Co^III^–corrinoids (Fig. 1 left) that catalyze biologically important methyl transfer and difficult [1, 2] rearrangement reactions.^1^ In MeCbl-dependent enzymatic reactions, the cobalt–carbon bond of MeCbl is cleaved heterolytically, which generates a square planar Cob(i)alamin species. Alternatively, in the case of AdoCbl-dependent enzymes, homolytic bond scission leads to a protein bound cofactor in the Co^II^ state and an organic 5′-deoxyadenosyl radical (Ado˙). This difference in organometallic reactivity is remarkable, considering that MeCbl and AdoCbl cofactors share identical features, as (i) the redox-active cobalt ion, (ii) the equatorially chelating corrin ligand, (iii) the lower coordinating dimethylbenzimidazole (Dmbz) base and (iv) the upper cobalt–carbon bond (Fig. 1, left).^2^,^3^ Obviously, a prominent role of the surrounding protein has been anticipated for triggering cobalt–carbon bond activation and enzymatic catalysis.^4^–^9^

**Fig. 1:**
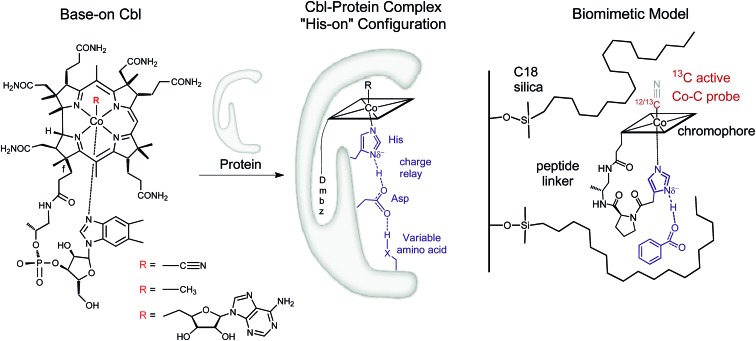
Left: Structure of vitamin B_12_ and Cbl cofactors having an intramolecularly bound dimethylbenzimidazole (“Dmbz”) (“base-on”) and distinct upper ligands (R = CN, B_12_; R = Me, MeCbl; R = 5′-deoxyadenosyl, AdoCbl). Middle: Cbl bound to the regulatory triad in the “histidine (“His”)-on” form, as found in several B_12_-dependent enzymes (X = donor atom, Table 1). Right: The corresponding biomimetic supramolecular model **1** with imidazole hydrogen-bonding interaction (charges are omitted).

In this context, most studies have focused so far on the impact of protein interactions with the upper axial ligand of the cofactor, whereas the interactions of the protein from the opposite site are less considered.^10^–^14^ Indeed, B_12_ cofactors are anchored to B_12_-dependent enzymes in two different ways: (i) the cofactor retains its intramolecularly coordinating Dmbz base (“base-on” form) during cofactor incorporation into the protein (*e.g.* class II ribonucleotide reductase or diol dehydratase) or (ii) the intramolecularly coordinating Dmbz base of the cofactor is replaced by a protein histidine (“His”) anchoring group (“His-on” configuration). This mode of protein-cofactor binding is shown in Fig. 1 (ref. 10) and encountered, amongst others, in MeCbl-dependent methionine synthase (MetH) as well as AdoCbl dependent methylmalonyl CoA mutase (MCM) and glutamate mutase (GluM) (Table 1).^15^–^17^

**Table 1:** Cbl ligation and lower part hydrogen bonding network in “His-on” configured B_12_ cofactor@protein complexes

Enzyme complex	Protein designation	Organism	Cofactor	Cofactor attachment and lower part hydrogen bonding network^*a*^	Reference
Methionine synthase	MetH	*Escherichia coli*, *Homo sapiens*	MeCbl	Asp-X-H[combining low line]i[combining low line]s[combining low line]-XX-Ser	18
Metyhmalonyl CoA mutase	MCM	*Propionibacterium shermanii*	AdoCbl	Asp-X-H[combining low line]i[combining low line]s[combining low line]-XX-Lys	19 and 20
		*Homo sapiens*, *Ascaris lumbricoides*			
Glutamate mutase	GluM	*Clostridium tetanomorphum*	AdoCbl	Asp-X-H[combining low line]i[combining low line]s[combining low line]-XX-Leu-X-Tyr^*b*^	21 and 22
		*Clostridium cochlearium*			

^*a*^The cobalt anchoring group is underlined.

^*b*^The Asp residue in GluM forms H-bonds with different residues (Leu and Tyr proposed here).

Furthermore, the Co-bound His is part of a hydrogen-bonded amino acid chain, together with an aspartic acid (Asp) and a variable third residue (lysine for MCM or serine for MetH, Table 1).^11^,^23^ The importance of the cofactor anchoring and the hydrogen bonding network for catalysis has been controversially discussed.^13^,^23^ For example, mutation studies with MetH indicated a dramatic loss of activity while replacing either His or Asp by other amino acids in the hydrogen bonded network.^23^ A different study focusing on “His-on” AdoCbl dependent isomerases indicate a structural reorganization of the hydrogen bonding network after substrate binding.^13^,^24^ This behavior is suggested to stabilize the catalytically active cob(ii)alamin intermediates by reducing the basicity of the anchoring His ligand.^13^ Such behaviour seems very appealing and could represent a general mode of remote control in reactions depending on “His-on” configured cofactor B_12_. Interestingly, a large number of heme-proteins such as globins, cytochromes c and horse radish peroxidase exhibit also a “His-on” type of cofactor attachment.^25^–^28^ Partial deprotonation of the proximal His ligand within a lower part hydrogen bonding network enables the heme-dependent enzymes to fine-tune their properties and reactivity (“push effect”).^29^–^33^ This type of redox control has been thoroughly investigated for heme proteins, as the Fe^III^–^13^CN protoporphyrin IX complexes,^34^–^36^ but was so far not studied for related mimics of “His on” Cbl@protein complexes.^12^,^13^,^24^,^37^–^44^ In this context, only one example reported so far the heterogeneous model of a Cob(iii)alamin–protein complex,^14^ whereas some fascinating immobilized models of heme–protein complexes have recently attracted considerable attention.^45^–^48^ Insights into the roles of the “His-on” cofactor attachment and the lower part hydrogen bonding network in B_12_-dependent enzymes are therefore still required. In this context, the development of molecular mimics,^13^,^40^,^42^ enzyme models^44^,^49^ and new supramolecular biomimetic architectures^14^ represent attractive assets.

We envisaged developing a new type of immobilized biomimetic model of the hydrogen bonding network, as found in “His-on” B_12_ cofactor complexes. This supramolecular assembly (Fig. 1 right) consists of three independent subunits: (i) a structurally modified metal cofactor complex, (ii) a carboxylate ion and (iii) a hydrophobic silica C18 solid support. In particular, we envisaged to induce intermolecular hydrogen bonding between the immobilized Co^III^-coordinated imidazole ligand and the carboxylate anion through hydrophobic interactions on the solid support.^30^

With this supramolecular immobilization strategy in mind, we began by developing the most important component of the supramolecular assembly: the molecular B_12_ derivative, mimicking the “His-on” Co^III^-complex. This intramolecular coordination compound consists of three subunits: (i) the tetradentate Co^III^–corrin complex, (ii) a lower (α-) coordinating imidazole unit and (iii) a *trans*-located cyanide ligand (Fig. 1, right). Such a molecular model allows a thorough study of the imidazole-deprotonation influence on the redox properties (cyclic voltammetry), on the chromophore (UV-vis) and on the *trans*-influence exercised on the upper (β-) axial CN group (^13^C-NMR). A meticulous structural modeling using QM/MM calculations (ESI, Fig. S1†) suggested the imidazole backbone derivative of B_12_**1-H^+^** as a close biomimetic of the “His-on” configuration in B_12_-cofactor protein complexes (Fig. 2).

**Fig. 2:**
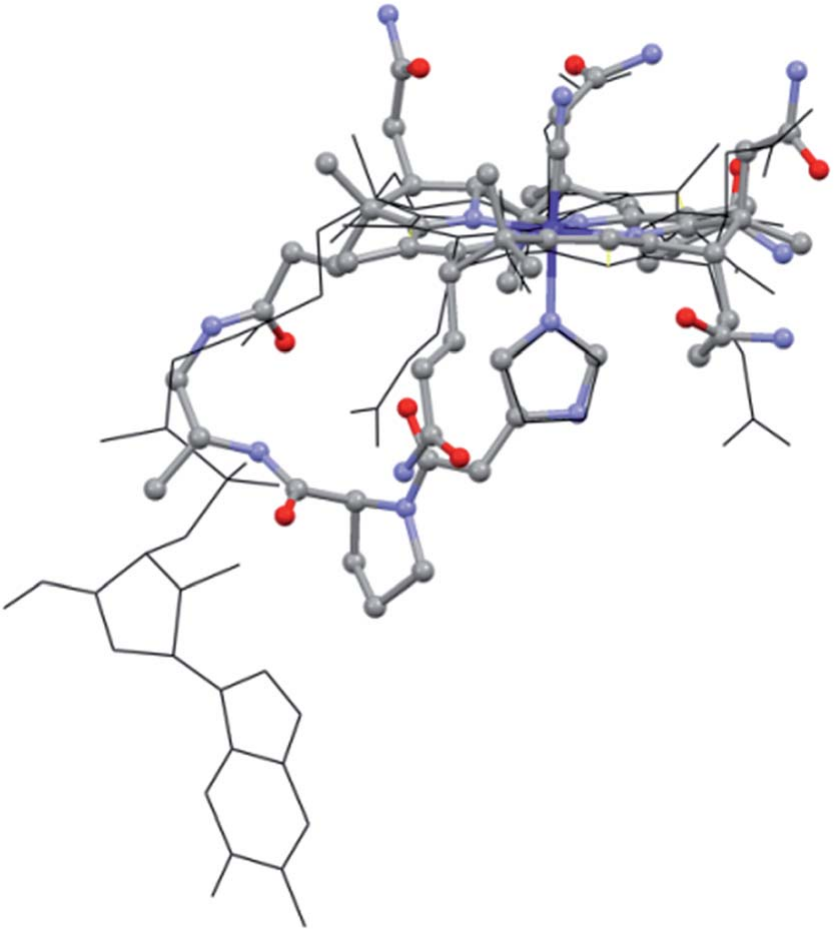
Overlap of the “His-on” configuration in a B_12_-cofactor protein complex (wireframe; protein: MetH, only MeCbl and His shown, PDB: ; 1BMT)^10^ and model of **1-H^+^** (ball and sticks).

In this compound, the imidazole subunit and the corrin macrocycle are connected *via* the *f*-side chain by an ethylenediamine spacer and a proline subunit.^50^–^52^ **1-H^+^** was synthesized as trifluoroacetic acid (TFA) salt in three steps with an overall yield of 19% using common peptide coupling chemistry (ESI, Schemes S1 and S2†). The UV-vis spectrum of **1-H^+^** resembles that of vitamin B_12_ (ESI, Fig. S2†), and the high resolution mass spectrum displays a signal at *m*/*z* = 610.30054 (calculated: *m*/*z* = 610.30032; [(**1-H^+^**) + H]^2+^).

The structural evaluation of the B_12_@protein model **1-H^+^** was investigated using 2D-ROESY. Through-space correlation was observed between H_im_2 (blue on Fig. 3) and the lower side chains 7A and 81 as well as the methyl group 51, all of which are situated at the northern face of the corrin ring. These interactions are complementary to correlations at the southern face of the molecule, between H_im_5 and the corrin side chains 151, 131, as well as the 1R situated on the peptide backbone (red on Fig. 3). These data thus unambiguously indicate a coordination of the imidazole group through the Nε-nitrogen. These measurements also allowed determining the positioning of the imidazole unit with respect to the macrocycle, and revealed that it follows the C51–C151 axis of the corrin ring (Fig. 3B). Furthermore, comparison with the active site of a “His-on” bound B_12_-cofactor from crystal structure indicates a good agreement with the structural behavior of **1-H^+^** (Fig. 2).

**Fig. 3:**
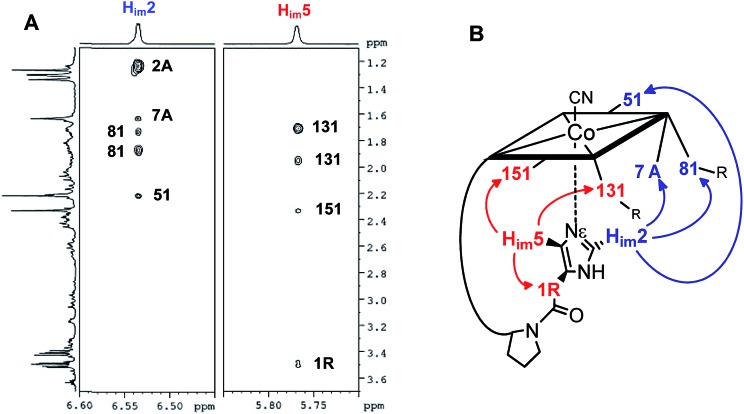
(A): 2D-ROESY coupling of **1-H^+^** (B): the corresponding correlation in the structure (in D_2_O, 270 K, 500 MHz) (charge is omitted).

Reversible proton release of **1-H^+^** (Scheme 1) was then tested by pH titration (from pH 8.5 to pH 12.5) and was detected with UV-vis spectroscopy. It revealed a bathochromic shift of 8 nm for both the α and β bands (ESI, Fig. S3†) which indicates clearly that upon proton release, the imidazole moiety transforms to a stronger σ-donating imidazolate ligand.^24^,^53^–^55^ Additionally, a p*K*_a_ value of 10.8 was determined for Nδ (ESI, Fig. S3†), which is about 4 pH units lower compared to free imidazole (p*K*_a_ = 14.5)^56^ and comparable to the values observed in His-anchored Fe^III^–^13^CN protoporphyrin IX@cytochrome *c* complexes (p*K*_a_ = 10.1 to 10.6).^36^

**Scheme 1:**
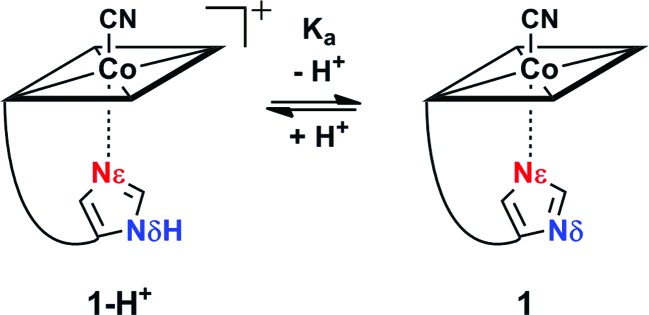
pH equilibrium between **1-H^+^** and **1**.

The electrochemical properties of the cobalt center in **1-H^+^** and **1** were examined with cyclic voltammetry.^57^–^59^ At pH 8.5, a cathodic peak at –920 mV *vs.* Ag/AgCl was observed for **1-H^+^** (Table 2, entry 2; Fig. S4†). We assign the cathodic wave to a two electron reduction of the octahedral coordinated Cob(III)alamin in a d^6^-electron configuration to a Co^I^-square planar complex in a d^8^-electron configuration, in agreement with electrochemical studies with B_12_.^57^,^60^ Switching from compound **1-H^+^** to **1** (pH 12.5) with a Co^III^-coordinated imidazolate ligand significantly shifted the cathodic peak by 180 mV to a value of –1100 mV *vs.* Ag/AgCl (Table 2, entry 1; ESI, Fig. S4†). Deprotonation of **1-H^+^** to **1** thus stabilizes the Co^III^ form against reduction.

**Table 2:** *E*_pc_, ^13^CN shift and position of the α-band of different B_12_ derivatives

Entry	Compound	*E*_pc_ ^*a*^ (mV)	CN ^13^C-NMR shift^*d*^ (ppm)	*λ* of α-band^*f*^ (nm)
1	**1**	–1100^*b*^	133	563
2	**1-H^+^**	–920^*b*^	127	556
3	B_12_	–897^*b*^	124	551
4	aquaCNCbi	–749^*c*^	112^*e*^	530
5	diCNCbi	–1170^*b*^	139^*e*^	580

^*a*^ *vs.* Ag/AgCl, 1.5 mM in 0.1 M KCl, scan rate: 0.005 V s^–1^.

^*b*^ *E*_pc_ for the Co^III^/Co^I^ reduction.

^*c*^ *E*_pc_ for the Co^II^/Co^I^ reduction.

^*d*^In D_2_O; 126 MHz.

^*e*^Values obtained from ref. 63.

^*f*^In H_2_O.

The influence of the axial ligand basicity on the *trans*-located β-CN moiety was then investigated with NMR spectroscopy. The measurements were performed at two different pH (pH 7.0 and 12.5), with an isotopically labeled ^13^CN ligand on the β-side of the cobalt. The ^13^C-NMR of **1-H^+^** revealed a broad signal at 127 ppm (^13^CN moiety) which shifts downfield (133 ppm) upon deprotonation (**1**) (ESI, Fig. S5†). This behavior is explained by the coordination of a stronger σ-donor (imidazolate, **1**), which results in a decreased polarization of the *trans*-located cyanide π-electron density.^61^,^62^ The broadening of the ^13^CN lines of **1** and **1-H^+^** (375 *vs.* 150 Hz) is due to the distinct nuclear quadrupolar properties of ^59^Co, which significantly increase the t1 and t2 relaxation rates of the directly bound carbon. These effects also prevent the observation of resolved ^13^C-multiplets due to scalar coupling between ^13^C and ^59^Co. Other broadening mechanisms like anisotropic effects or paramagnetic impurities can be excluded, because all remaining ^13^C resonances of the measured compounds are sharp (ESI, Fig. S5†).

Control experiments (CV, UV-vis, ^13^C-NMR) were performed with a ‘blocked’ model at pH 7.0 and pH 12.5 in the same manner described for **1-H^+^** and **1** (ESI, Fig. S6†). B_12_ was used for this purpose, since it contains an α-coordinating Dmbz ligand resembling the imidazole unit, for which reversible protonation of both nitrogens is impossible (Fig. 1, left). In effect, all control experiments with B_12_ exhibited no significant shifts upon pH changes, thus proving that all results obtained with **1-H^+^** and **1** originated from the deprotonation of the imidazole ligand.

Plotting the position of the cathodic peak *E*_pc_ against the upper cyanide ^13^C-NMR shift for the ‘blocked’ model B_12_, the biomimetic compounds **1** and **1-H^+^** as well as two other compounds which present distinct lower axial ligand (aquacyanocobinamide, aquaCNCbi; Fig. 4, right; and dicyanocobinamide, diCNCbi; Fig. 4, right) gives a more extensive picture on the influence of lower ligand modulation (Fig. 4).^13^,^24^,^64^,^65^ The linear correlation suggests that the relative σ-donating capacities of the lower ligand similarly influence both the cathodic peak (*E*_pc_) and the upper CN ^13^C shift. Furthermore, by considering that aquaCNCbi represents a model for base-off B_12_, important insights are extracted on how Cbls and Cbl@protein complexes may alter their electrochemical properties in biological systems. Indeed, when considering B_12_ alone, the tuning of the redox properties is reached only through the “base-on/base-off” switch, where the α-Dmbz ligand is replaced by the weaker electron donating H_2_O molecule. However, this modification only leads to the destabilization of the cobalt center, as shown by the less negative *E*_pc_ value for the Co(ii)/Co(i) reduction of aquaCNCbi (*E*_pc_ = –749 mV *vs.* Ag/AgCl, Table 2, entry 4). The contrary effect, a stabilization of the metal ion seems not to be possible in this coordination mode. Switching the α-Dmbz ligand of B_12_ to an imidazole moiety, as encountered in **1-H^+^**, exhibits only a small effect on the Co^III^/Co^I^ reduction (Δ*V* = –23 mV, Table 2, entry 2 and 3). However, deprotonation to an imidazolate moiety (**1**) is now possible and leads to a substantial cathodic shift of –203 mV (Table 2, entry 1–3). The lower ligand modulation of **1-H^+^** thus leads to a doubling of the available redox range (Fig. 4, –749 to –1100 mV *vs.* Ag/AgCl) compared to B_12_ alone (Fig. 4, –749 to –897 mV *vs.* Ag/AgCl). This behavior underscores strikingly that (partial) deprotonation of the coordinated His-residue offers B_12_-dependent proteins an elegant tool for fine-tuning their electron donating properties on demand. The lower ligand modulation is also reflected in a bathochromic shift of the α-band (Table 2) and the β-band wavelengths (data not shown). Furthermore, linear correlations are observed between the *E*_pc_ and the α-band (Fig. 4) or the β-band (data not shown). This behavior is in line with recent spectroscopic and theoretical studies, which show that the position of the α-band reflects the d_*z*2_ contribution of the lower coordinating ligand to the HOMO orbital.^24^,^53^–^55^

**Fig. 4:**
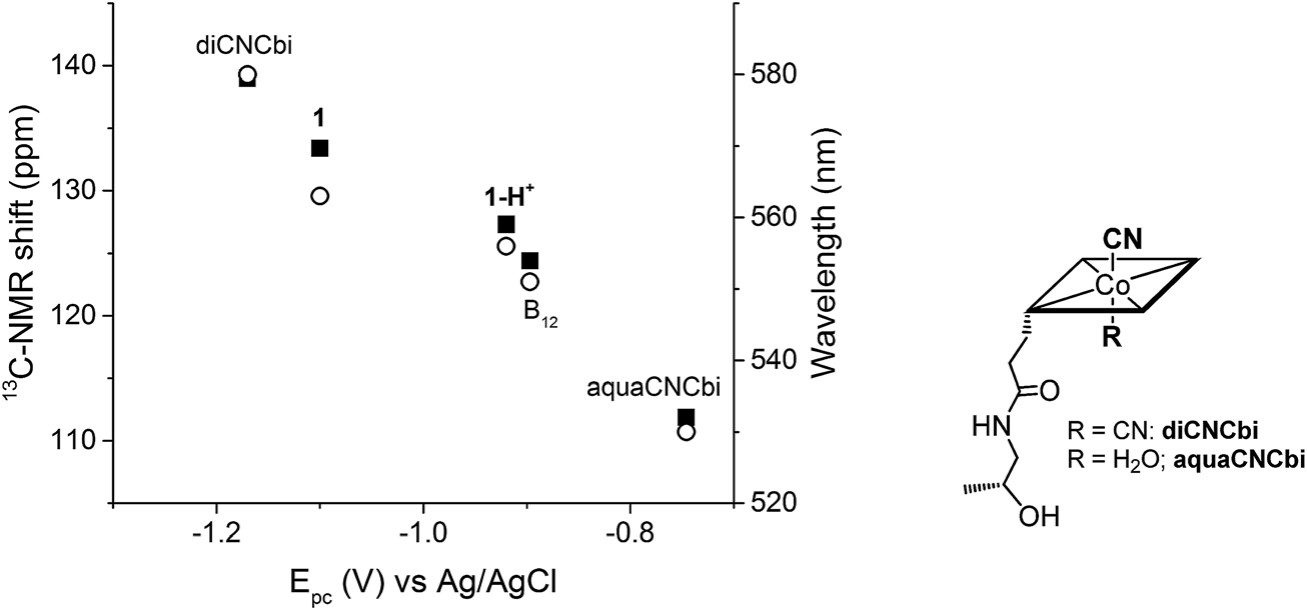
Left: Correlation between *E*_pc_ (Co^III^/Co^I^ reduction for all compounds, except aquaCNCbi: Co^II^/Co^I^ reduction) and the ^13^C-NMR shift of CN (left axis, ■) and the α-band wavelength (right axis, ○); B_12_ data correspond to both pH 7.0 and pH 12.5. Right: Structure of aquaCNCbi and diCNCbi (charges are omitted).^66^

In His-on Cbl complexes, the deprotonation of the His residue is achieved through an H-bonded chain in between the ligand triad. However, under aqueous conditions, deprotonation was only achieved at pH values above the p*K*_a_ of **1-H^+^** (10.8). For mimicking the biological situation with the model **1-H^+^** more closely, we thus decided to investigate the interactions between the imidazole of **1-H^+^** and an exogenous carboxylate as H-bonding acceptor. For this purpose, we applied first benzoic acid in dioxane–H_2_O mixtures of **1-H^+^**,^67^ which did not result in any spectroscopic changes. Immobilized **1-H^+^** (**1-H^+^_SP_**) on C18 silica material behaved differently and allowed studying proton release with diffuse reflectance spectroscopy, an approach recently introduced by our group.^14^

**1-H^+^_SP_** remains base-on upon immobilization, as indicated by the characteristic reflectance spectrum of a “base-on” Cbl (ESI, Fig. S7†). In agreement with studies under homogenous conditions, treatment of **1-H^+^_SP_** with a strong base (1 M NaOH) led to a bathochromic shift (6 nm for the α-band) and indicated imidazole deprotonation (Fig. 5; ESI, Fig. S7†). However, the addition of pure toluene, which lacks any H-bond acceptor, to **1-H^+^_SP_** did not lead to any changes. In contrast, the further addition of benzoate (tetrabutylammonium salt) to this solvent led to a slight, but characteristic bathochromic shift of the α-band (4 nm) and β-band (2 nm) (Fig. 5; ESI, Fig. S8†), which suggests hydrogen-bonding between benzoate and the Nδ hydrogen of **1-H^+^_SP_**, similar to the situation proposed for this class of proteins.^10^,^13^

**Fig. 5:**
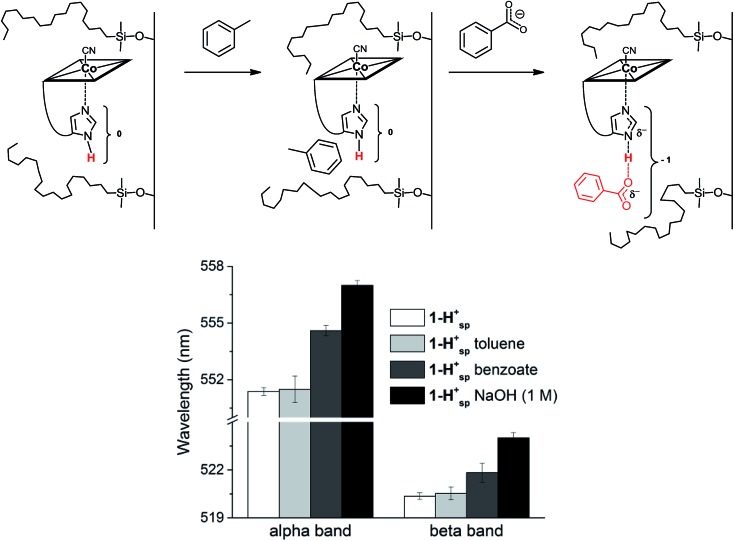
Top: Proposed mechanism for the reaction of **1-H^+^_SP_** immobilized on C18 silica (left) with toluene (middle) and with benzoate (tetrabutylammonium salt; right). Bottom: Absorbance for the α and β bands of immobilized **1-H^+^_SP_** (white), **1-H^+^_SP_** after the addition of toluene (light grey), **1-H^+^_SP_** after the addition of a solution of benzoate in toluene (dark grey) and **1-H^+^_SP_** after the addition of 1 M NaOH (black) (*n* = 4) (charges are omitted).

In summary, we present an unprecedented supramolecular assembly mimicking the regulatory ligand triad of “His-on” metal-cofactor@protein complexes. The data presented herein give evidence that deprotonation of an axially coordinating imidazole to form imidazolate stabilizes significantly (Δ*V* = –203 mV) the Co^III^ form of the corrinoid. However, such behavior cannot be achieved with B_12_ in its base-on form. Modulating the corrinoid redox properties with this elegant trick represents therefore a sophisticated strategy for tailoring the electronic properties of Cbls in biological systems.

## Supplementary Material

Supplementary information
