# Supplementary material for: Modulating the cobalt redox potential through imidazole hydrogen bonding interactions in a supramolecular biomimetic protein-cofactor model
Source: Chem Sci. 2016 Feb 23;7(6):3836–42. doi: 10.1039/c5sc04396d (PMC6013808; doi:10.1039/c5sc04396d)
Supplement: Supplementary file 1 [file SC-007-C5SC04396D-s001.pdf]

Electronic Supplementary Material (ESI) for Chemical Science.
This journal is © The Royal Society of Chemistry 2016

Supporting Information for

Modulating the Cobalt Redox Potential through Imidazole Hydrogen Bonding Interactions in a Supramolecular Biomimetic Protein-Cofactor Model

Marjorie Sonnay, Thomas Fox, Olivier Blacque and Felix Zelder*

Contents

Material and Methods 2

Synthetic Schemes S1, S2 3

Experimental Procedure for compounds 1 - 9 3

QM-MM Calculations 6

Figure S1: ONIOM layer definition used for the QM/MM geometry optimization of **1-H⁺** 6

Figure S2: Absorbance spectra of **1-H⁺** and vitamin B_{12} 6

Figure S3: pH titration of **1-H⁺** and corresponding fitting 7

Figure S4: CV of **1-H⁺** and **1** 7

Figure S5: CN ^{13}C-NMR of **1-H⁺** and **1** 7

Figure S6: CV, absorbance spectrum and CN ^{13}C-NMR of B_{12} at pH 7.0 and pH 12.5 8

Figure S7: DRUV-vis of immobilized $\mathbf{1\text{-}H^+_{sp}}$ and after addition of 1M NaOH solution 8

Figure S8: DRUV-vis of immobilized $\mathbf{1\text{-}H^+_{sp}}$ and after addition of toluene and a saturated benzoate solution in toluene 9

Figure S9 – ^{1}H-NMR of **4** 9

Figure S10 – ^{1}H-NMR of **5** 9

Figure S11 – ^{1}H-NMR of **1-H⁺** 10

Table 1: Cartesian coordinates of the QM/M optimized **1-H⁺** 10

References 11

Materials and Methods

All chemicals were of reagent grade and used without further purification. Deuterated solvents were obtained from Armar Chemicals (Döttingen, Switzerland). Solvents for HPLC and UPLC were of the corresponding grade. Dicyanocobyric acid (**2**) was prepared from the method of Müller *et al.*[S1] The dicyano derivatives **4** and **5** were obtained by addition of 1 eq of KCN after purification of the compounds. Compounds **1-H$^+$** was obtained as trifluoroacetate (TFA) salt after preparative HPLC.

HPLC solvents were 0.1% aqueous trifluoroacetic acid (A) and methanol (B) or acetonitrile (C). Analytical HPLC analyses were performed on a Merck-Hitachi L-7000 system equipped with a diode array UV-Vis spectrometer. Macherey Nagel Nucleosil C18ec RP column (5 µm particle size, 100 Å pore size, 250 x 3 mm. Flow rate: 0.5 ml min^{-1}) was used as column. Preparative HPLC separations were performed on a Varian Prostar system equipped with two Prostar 215 pumps, a Prostar 320 UV/Vis detector and Macherey Nagel Nucleosil C-18ec RP column (7 µm particle size, 100 Å pore size, 250 x 21 mm. Flow rate: 18 ml min^{-1}) was used as column. Preparative gradients are: gradient 1 (0-5 min: 25% B; 5-30: 25-100%B); gradient 2 (0-3 min: 3%C. 3-35 min: 3-30%C).

Solid phase extraction (SPE) was conducted using Chromafix C18ec by Macherey-Nagel. The sample was dissolved in water and charged on the preconditioned absorbent. After washing with water, the compound was eluted with MeOH.

UV-Vis spectra were recorded on a Cary 50 spectrometer using quartz cells with a path length of 1 cm. The pK_a values were obtained from the analysis of a Boltzmann function: y = A2+ (A1 - A2) / (1 + exp((x - x0) / dx)).

DRUV/Vis spectra were recorded with a Lambda 650 S Perkin-Elmer UV/Vis spectrometer in the range 300–700 nm using a Quartz SUPRASIL precision cell (10 mm). Sample preparation was performed as described elsewhere.[S2]

NMR spectra were recorded on a Bruker AV-500 spectrometer (Karlsruhe, Germany). For ^{1}H-NMR, the chemical shifts are given in ppm relative to the signal from the deuterated solvent. Coupling constants J are given in Hz. For ^{13}C-NMR, the chemical shifts are given in ppm relative to (trimethylsilyl)propionic acid sodium salt. Experiments at different pH were performed in 0.1 M phosphate buffer in D_2O (pH = 7.0) and pH 12.5 was obtained by adding ~1 µl of NaOD in 0.5 ml D_2O.

Mass spectra were recorded in the positive mode on an Esquire HCT from Bruker (Bremen, Germany). The injection rate was 3 µL/min. A nebulizer pressure of 10 psi, a dry gas flow rate of 5 l/min and a gas temperature of 350°C were applied. All solvents were of LCMS grade.

High-resolution electrospray mass spectra were recorded on a Bruker maXis QTOF-MS instrument (Bruker Daltonics GmbH, Bremen, Germany). The samples were dissolved in MeOH and analyzed via continuous flow injection at 3 µL/min. The mass spectrometer was operated in positive ion mode with a capillary voltage of 4 kV, an endplate offset of –500 V, nebulizer pressure of 5.8 psig, and a drying gas flow rate of 4 l/min at 180°C. The instrument was calibrated with a sodium formate solution (500µl H_2O : 500µl iPrOH : 20ul HCOOH : 20µl 0.1M NaOH aq). The resolution was optimized at 30’000 FWHM in the active focus mode. The accuracy was better than 2 ppm in a mass range between m/z 118 and 1600. All solvent used were purchased in best LCMS qualities.

Cyclic voltammetry was conducted on a 757 VA computrace electrochemical analyzer (Ω, Metrohm). Ag/AgCl electrode was used as reference and glassy carbon as working electrode. The samples (1.5 mM) were dissolved in 0.1 M KCl (2 ml), and $K_3Fe(CN)_6$ (1 mM) was used as internal reference. Before each measurement, the glassy carbon electrode was polished for 1 minute and the sample was purged with nitrogen for 3 min. Sweep rate: 0.005 V/s.

Scheme S1 - Synthesis of **6** (A: HOBt, EDC, DIPEA, DMF, 0°C. B: 0.5M NaOH)

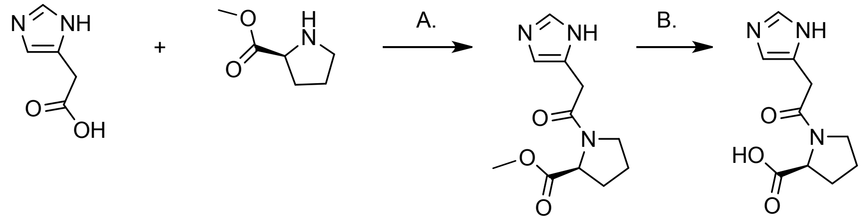

7 **8** **9** **6**

Scheme S2 -. Synthesis of **1-H⁺** (A. NEt_3, ethylchloroformate, DMF, 0°C. B. HCl 1M. C. DIPEA, HOBt, TBTU, DMF, 0°C)

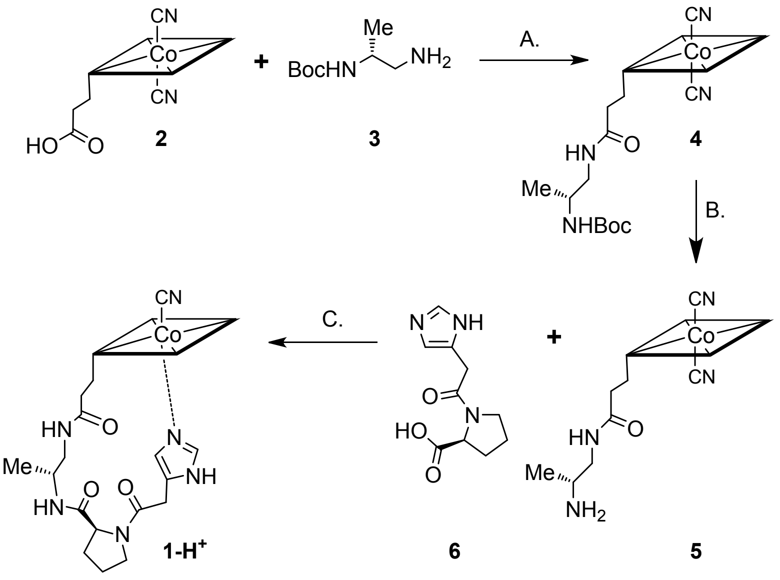

Experimental Procedures:

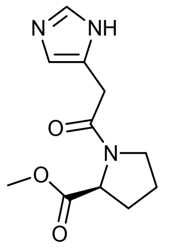

Compound 9: Imidazole acetic acid (**7**) (200 mg, 1.23 mmol) was dissolved in dry DMF (8 ml) and cooled to 0°C. L-proline (**8**) (192 mg, 1.23 mmol) and HOBt (240 mg, 1.35 mmol) were then added. After 10 min, EDC (248 mg, 1.35 mmol) and DIPEA (214 µg, 1.26 mmol) were added, and the reaction mixture was let stir overnight. The solution was added to 20 ml of a saturated $NaHCO_3$ water solution and extracted with 4 portions of 30 ml of dichloromethane (DCM). The organic phase was dried on $MgSO_4$. After filtration, the compound was purified by flash chromatography on silica gel (EA/DCM/MeOH/NH_3 16:24:10:1.5). The solvent was removed under reduced pressure, to obtain **9** as transparent oil (213.7 mg, yield: 75 %).

TLC (Silicagel 60 F_{254}, EA/DCM/MeOH/NH_3 16:24:10:1.5): R_f = 0.53

HR-MS: $[M+H]^+$ calculated for $C_{11}H_{16}O_3N_3$: 238.11862, found 238.11821.

^{1}H-NMR : (D_2O, 500 MHz) δ /ppm = 7.97 (s, 1H), 7.13 (s, 1H), 4.50 (dd, J = 8.5, 4.0 Hz, 1H), 3.83 (d, J = 1.8 Hz, 2H), 3.77 (s, 3H), 3.76 – 3.51 (m, 2H), 2.43 – 2.27 (m, 1H), 2.11 – 1.99 (m, 3H). (a ratio of the syn / anti isomers of approximately 83:17 was calculated. Only the major isomer is reported).

^{13}C-NMR : (D_2O, 500 MHz) δ /ppm = 184.16, 177.73, 177.21, 174.47, 173.98, 167.80, 138.36, 132.54, 120.10, 63.13, 62.28, 56.05, 55.77, 50.80, 49.86, 35.42, 34.96, 34.23, 33.59, 31.84, 27.18, 24.83. (two isomers present).

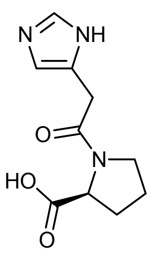

Compound 6: 9 (100 mg, 0.42 mmol) was dissolved in 0.5M NaOH, and let react for 2h. The pH was then set to 5 with a 1M HCl solution, and the solvent was removed under reduced pressure. The salts were then removed by precipitation in 5 ml of MeOH and filtered off. After removal of the solvent under reduced pressure, 90.2 mg of transparent oil was collected (yield: 98%).

HR-MS: $[M+H]^+$ calculated for $C_{10}H_{14}O_3N_3$: 224.10297, found 224.10325.

^{1}H-NMR : (D_2O, 400 MHz) δ / ppm = 8.75 (s, 1H), 7.47 (s, 1H), 4.37 (dd, J = 8.9, 4.0 Hz, 1H), 4.07 (s, 2H), 3.78 – 3.67 (m, 1H), 3.63 – 3.47 (m, 1H), 2.43 – 2.23 (m, 2H), 2.08 – 1.96 (m, 1H), 1.97 – 1.79 (m, 1H). (a ratio of the syn / anti isomers of approximately 60:40 was calculated. Only the major isomer is reported)

^{13}C-NMR : (D_2O, 500 MHz) δ / ppm = 181.69, 181.16, 172.08, 171.69, 136.37, 136.34, 129.64, 120.53, 65.39, 63.95, 51.84, 50.88, 50.29, 34.43, 33.05, 32.82, 32.47, 27.27, 25.47, 24.25. (two isomers present).

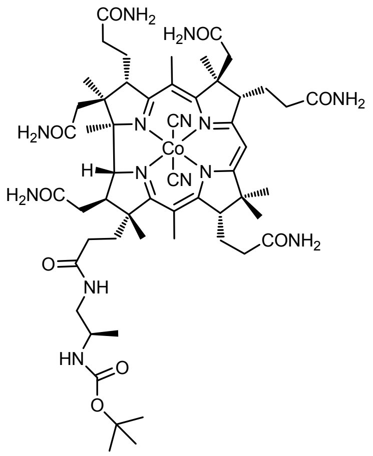

Compound 4: Cobyric acid **2** (20 mg, 18.2 µmol) was dissolved in dry DMF (8 ml), then NEt_3 (18 µl, 133.3 µmol) and ethylchloroformate (10 µl, 105.0 µmol) were added. After 5 min, (R)-tert-Butyl (1-aminopropan-2-yl)carbamate **3** (20.7 mg, 111.6 µmol) was added. The reaction was quenched with water (15 ml) after 15 min, and washed with 3 portions of DCM (30 ml). The collected water phase was then purified by preparative HPLC (gradient 1) to afford **4** (12.1 mg, yield: 52 %).

HR-MS: $[M\text{-}CN]^+$ calculated for $C_{54}H_{81}CoN_{13}O_9$: 1114.56067, found 1114.56100.

UV-Vis of **4**: (H_2O) λmax / nm (log ε)= 580 (4.00); 539 (3.94); 368 (4.48); 312 (3.98); 277 (3.98).

^{1}H-NMR of **4**: (D_2O, 500 MHz) δ / ppm = 5.96 (s, 1H), 3.92 (d, J = 8.5 Hz, 1H), 3.82 (d, J = 10.5 Hz, 2H), 3.47 (dd, *J* = 7.0, 4.9 Hz, 1H), 3.37 (d, J = 5.8 Hz, 2H), 3.28 - 3.04 (m, 2H), 2.96 (s, 1H), 2.85 – 2.72 (m, 2H), 2.70 - 2.58 (m, 3H), 2.58 - 2.45 (m, 2H), 2.45 – 2.37 (m, 6H), 2.37 – 2.29 (m, 6H), 2.29 - 2.14 (m, 3H), 2.14 - 2.00 (m, 2H), 1.98 - 1.80 (m, 3H), 1.76 (s, 3H), 1.60 (s, 3H), 1.55 (s, 3H), 1.51 (s, 3H), 1.48 (s, 9H), 1.38 (s, 3H), 1.26 (s, 3H), 1.17 (d, J = 6.8 Hz, 3H).

^{13}C-NMR of **4**: (D_2O, 500 MHz) δ / ppm = 181.5, 181.4, 180.9, 180.4, 180.1, 179.5, 178.7, 178.6, 178.0, 177.9, 174.8, 166,2, 165.8, 122.6, 120.3, 118.0, 115.7, 107.9, 105.9, 93.8, 85.9, 83.5, 77.7, 73.9, 61.6, 59.0, 58.0, 56.0, 52.0, 49.6, 49.2, 49.1, 48.8, 47.3, 46.6, 45.1, 41.6, 37.7, 35.6, 34.7, 34.6, 34.3, 33.3, 30.5, 29.4, 28.1, 27.6, 24.5, 21.5, 21.3, 20.4, 20.1, 19.2, 17.9, 17.6.

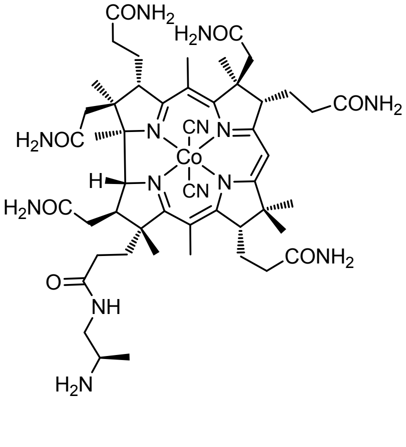

Compound 5: 4 (20 mg, 15.9µmol) was dissolved in 1M HCl (6 ml) and let stirred for 1 hour. The reaction mixture was neutralized and the solvent was removed under reduced pressure. The mixture was purified by preparative HPLC (gradient 2) to afford **5** (15.6 mg, yield: 85%).

HR-MS: $[M\text{-}CN]^+$ calculated for $C_{49}H_{73}CoN_{13}O_7$: 1014.50824, found 1014.50900.

UV-Vis of **5**: (H_2O) λmax / nm (log ε) = 579 (4.02), 539 (3.96), 417 (3.38), 367 (4.48), 307 (4.02), 276 (4.05).

^{1}H-NMR of **5**: (D_2O, 500 MHz) δ / ppm = 5.93 (s, 1H), 3.87 (d, J = 8.5 Hz, 1H), 3.78 (d, J = 10.4 Hz, 1H), 3.44 (dd, J = 7.0, 4.9 Hz, 1H), 3.33 (d, J = 14.0 Hz, 4H), 3.24 (s, 1H), 3.00 – 2.90 (m, 1H), 2.76 (dd, J = 10.7, 6.3 Hz, 2H), 2.68-2.54 (m, 3H), 2.54 – 2.45 (m, 2H), 2.39 – 2.33 (m, 6H), 2.33 – 2.26 (m, 6H), 2.26 – 2.09 (m, 3H), 2.09 - 1.97(m, 1H), 1.96 - 1.75 (m, 3H), 1.72 (s, 3H), 1.57 (s, 3H), 1.53 (s, 3H), 1.47 (s, 3H), 1.35 (s, 6H), 1.27 – 1.16 (m, 2H).

^{13}C-NMR of **5**: (D_2O, 500 MHz) δ / ppm = 181.6, 181.4, 180.9, 180.4, 180.2, 179.7, 178.8, 178.1, 178.1, 174.9, 166.3, 166.0, 165.8, 165.7, 120.4, 118.0, 108.0, 105.9, 93.9, 85.9, 77.9, 61.6, 59.1, 58.1, 56.0, 52.0, 49.6, 49.1, 48.9, 46.5, 45.1, 41.8, 37.7, 35.2, 34.7, 34.6, 34.3, 33.2, 29.5, 28.5, 27.7, 24.5, 21.5, 21.4, 21.3, 20.1, 19.3, 18.0, 17.6.

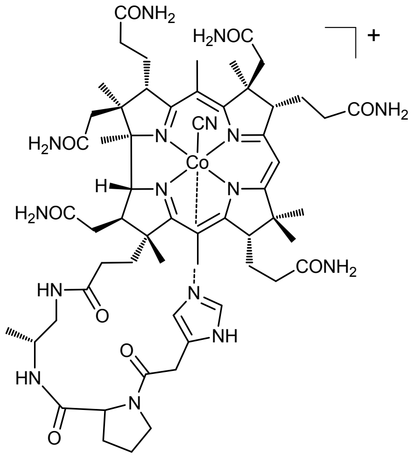

Compound 1-H⁺ (TFA salt): **6** (12 mg, 53.3 µmol) was dissolved in dry DMF (4 ml) and cooled to 0°C. HOBt (6.6 mg, 48.8 µmol), TBTU (15.6 µl, 48.8 µmol) and DIPEA (68 µl, 399.9 µmol) were then added. After 5 minutes, the solution was added to a solution of **5** (20 mg, 17.3 µmol) dissolved in dry DMF (5 ml). The mixture was stirred for 18h. The reaction was then quenched with water (15 ml) and washed 3 times with DCM (20 ml). The water phase was further purified by preparative HPLC (gradient 1), which afforded a mixture of the base-on and base-off forms of **1-H⁺** (10.5 mg, yield: 45 %). To obtain the base-on form of **1-H⁺**, the compound was dissolved in water (6 ml) and converted with KCN ($K^{12}CN$ or $K^{13}CN$) to the corresponding dicyano-derivative. Afterward, the pH was lowered to 3.8 with 1M HCl, and the reaction was stirred at room temperature for 24h. The solvent was then removed under reduced pressure to afford a pink powder.

HR-MS: $[M+H]^{2+}$ calculated for $C_{59}H_{85}CoN_{16}O_9$: 610.30032, found 610.30054.

UV-Vis: (H_2O) λmax / nm (log ε) = 556 (3.99); 522 (3.95); 362 (4.49); 323 (3.87); 306 (3.94); 277 (4.09).

^{1}H-NMR of **1-H⁺**: (D_2O, 500 MHz) δ / ppm = 6.64 (s, 1H), 6.07 (s, 1H), 5.94 (s, 1H), 4.27 (dd, J = 8.5, 4.6 Hz, 1H), 4.24 - 4.13 (m, 1H), 4.09 – 4.00 (m, 2H), 3.65 – 3.56 (m, 4H), 3.48 (dd, J = 10.2, 4.8 Hz, 2H), 3.37 – 3.27 (m, 1H), 2.90 - 2.67 (m, 3H), 2.67 – 2.52 (m, 5H), 2.52 - 2.47 (m, 5H), 2.42 – 2.30 (m, 15H), 2.30 – 2.14 (m, 4H), 2.13 – 1.97 (m, 3H), 1.96 – 1.85 (m, 4H), 1.85 – 1.79 (m, 3H), 1.50 (s, 3H), 1.48 (s, 3H), 1.47 (s, 3H), 1.41 (s, 3H) 1.19 (s, 3H), 1.16 (s, 3H), 0.69 (s, 3H). (A ratio of syn/anti rotamers of approximately 88:12 originating from restricted rotation around the tertiary amide bond of the proline subunit was calculated from signals at 5.94 ppm and 5.81 ppm.[S3-S5])

^{13}C-NMR of **1-H⁺**: (D_2O, 500 MHz) δ / ppm = 181.9, 181.7, 181.6, 181.3, 181.2, 180.4, 179.2, 179.0, 178.4, 177.4, 172.1, 168.5, 166.6, 166.1, 137.5, 134.2, 128.6, 127.5, 125.7, 120.9, 118.6, 113.0, 112.8, 109.3, 106.8, 96.9, 87.7, 78.3, 65.8, 63.7 61.9, 59.3, 58.7, 56.9, 53.8, 51.5, 51.1, 50.1, 48.1, 47.5, 46.5, 42.3, 38.0, 36.9, 36.2, 35.4, 35.3, 34.5, 33.4, 33.1, 30.6, 29.4, 28.4, 27.5, 22.2, 21.0, 20.1, 20.0, 19.8, 18.6, 18.0, 17.5.

QM/MM Calculations - The geometry of the cobalamin molecule **1-H⁺** was optimized with a combined quantum mechanics/molecular mechanics (QM/MM) calculation using the Gaussian09's two-layer ONIOM method.[S6-10] **1-H⁺** was partitioned into two regions as illustrated in Figure S1. The High Layer corresponds to the smallest part and was treated at the DFT level with the popular B3LYP (Becke, three-parameter, Lee-Yang-Paar) exchange-correlation functional [S11-S12] in conjunction with the LANL2DZ [S13-16] basis set. In a two-layer model, the Low Layer consists of the entire molecule. The corresponding low layer atoms were treated with the UFF force field.[S17] The geometry was then further optimized, using the 2D-ROESY results.

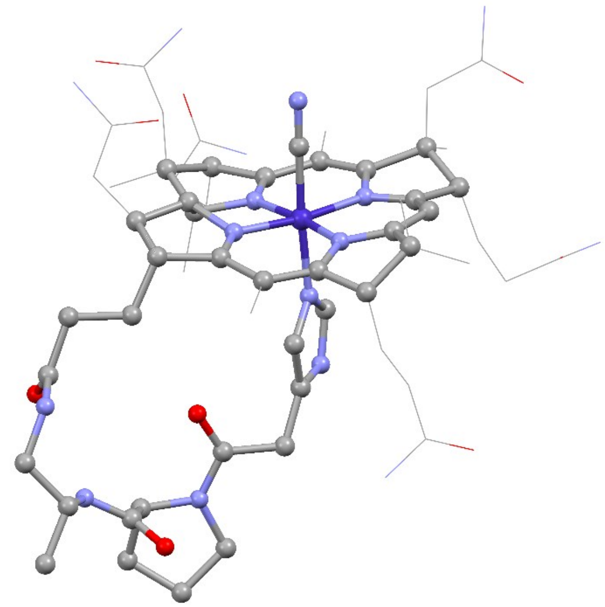

Figure S1 - ONIOM layer definition used for the QM/MM geometry optimization of **1-H⁺** (QM: balls and sticks, MM: wireframe).

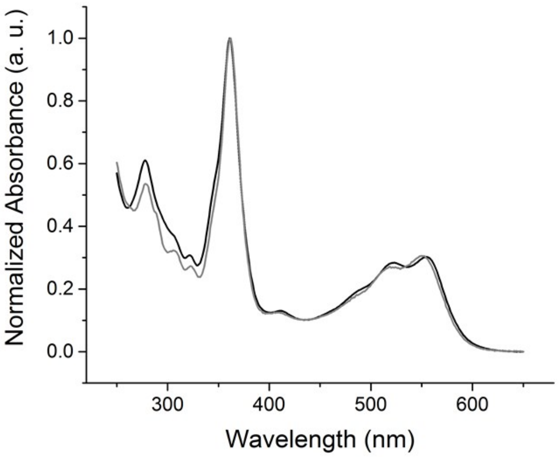

Figure S2 – Absorbance spectra of **1-H⁺** (black) and vitamin B_{12} (grey).

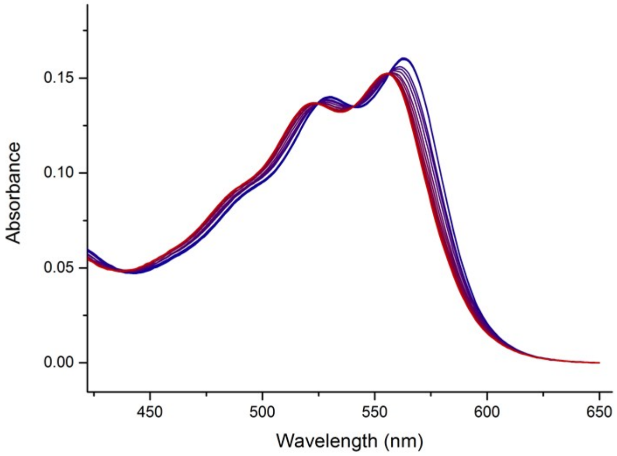

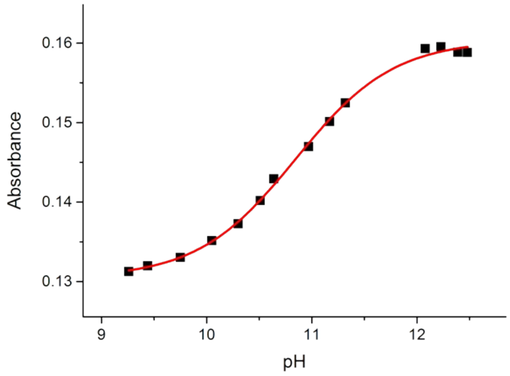

Figure S3 – Left: pH titration of **1-H⁺** from pH 8.5 (red) to pH 12.5 (blue); Right: corresponding pka determination plot (absorbance at 565 nm).

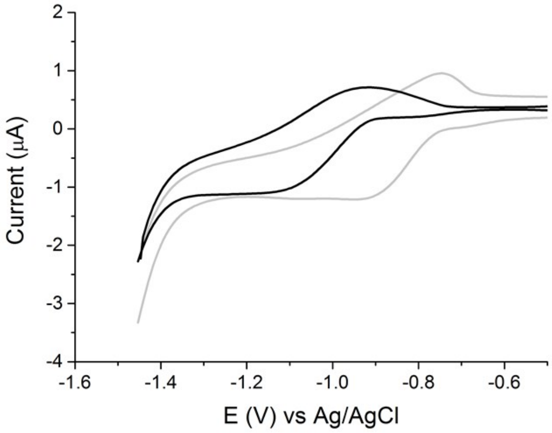

Figure S4 - CV of **1-H⁺** (pH = 8.5, grey) and **1** (pH = 12.5, black).

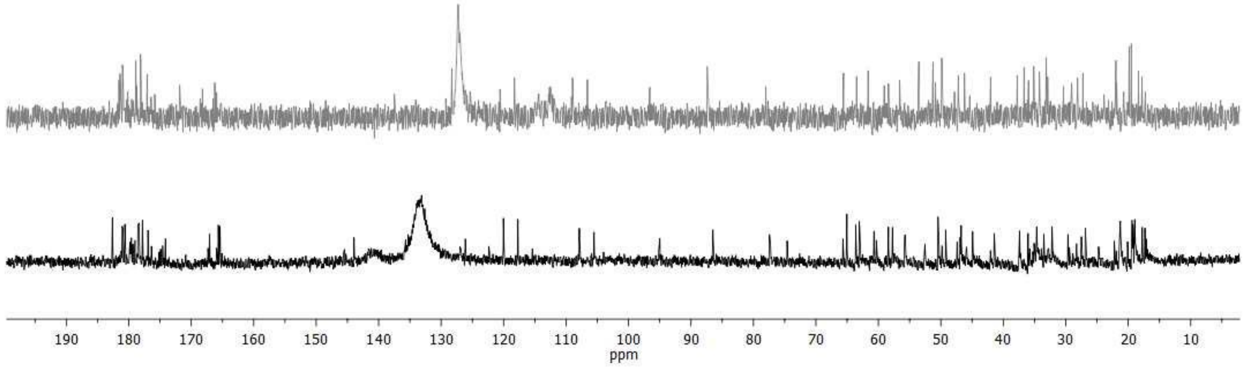

Figure S5 – ^{13}C-NMR spectra of **1-H⁺** (pH = 7.0, grey) and **1** (pH = 12.5, black), D_2O (pH = 7.0: 0.1 M phosphate buffer, pH = 12.5: ~1 μl of NaOD in 0.5 ml D_2O; 126 MHz).

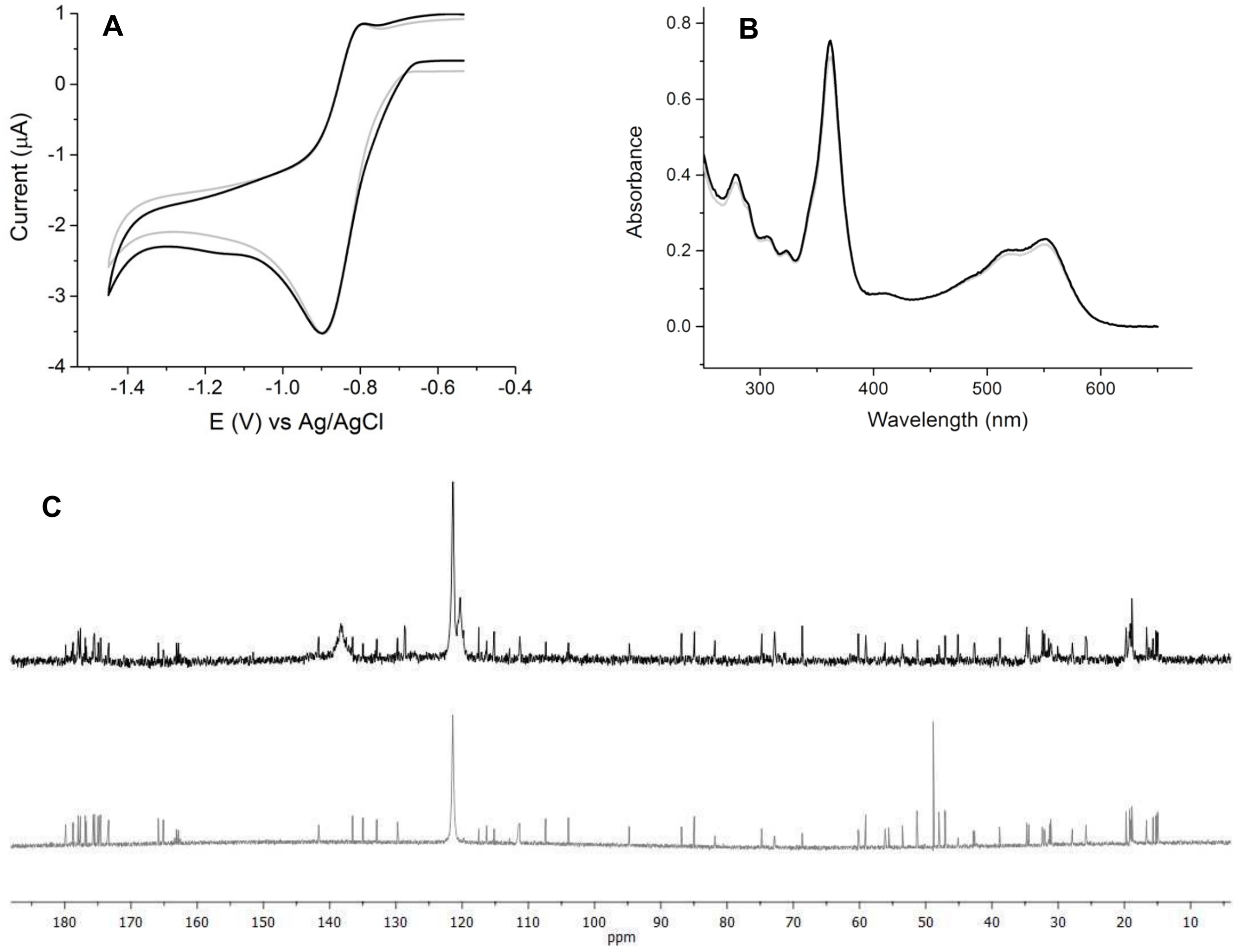

Figure S6 - **A**: CV of B_{12} at pH 7.0 (grey) and pH 12.5 (black). **B**: Absorbance spectra of B_{12} at pH 7 (grey) and pH 12.5 (black). **C**: ^{13}C-NMR spectra of B_{12} at pH 7.0 (grey) and pH 12.5 (black), D_2O (pH = 7.0: 0.1 M phosphate buffer, pH = 12.5: ~1 µl of NaOD in 0.5 ml D_2O; 126 MHz).

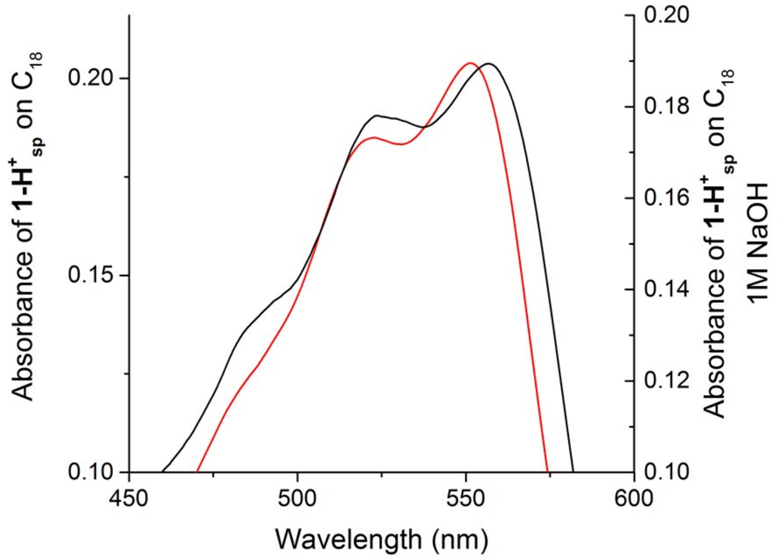

Figure S7 - DRUV-vis of **1-H^+_{sp}** (red) and **1-H^+_{sp}** after addition of 1M NaOH solution (black).

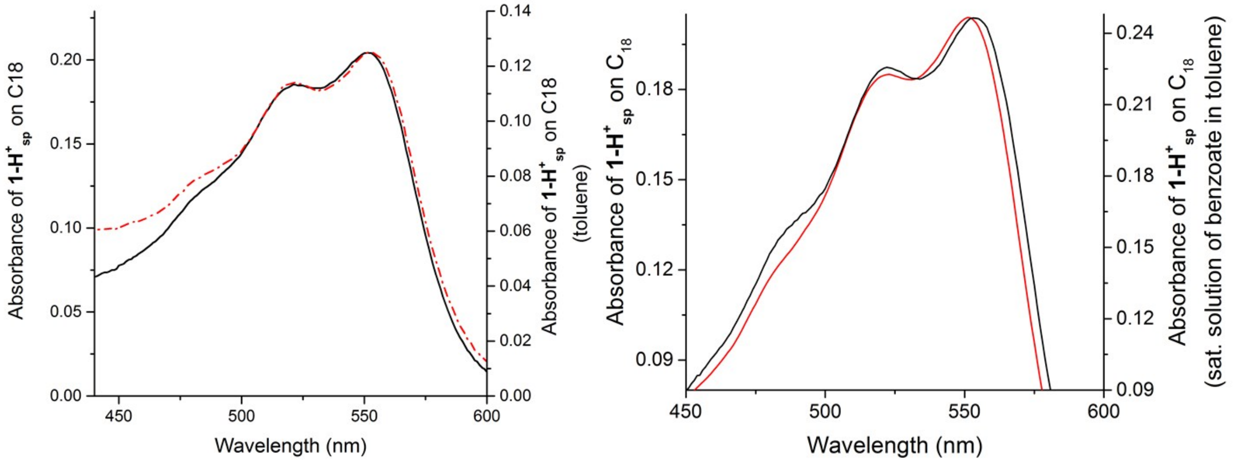

Figure S8 - Left: DRUV-vis of **1-H⁺sp** (black) and **1-H⁺sp** after addition of toluene (red). Right: **1-H⁺sp** (red) and **1-H⁺sp** after addition of a saturated tetrabutylammonium benzoate solution in toluene (black).

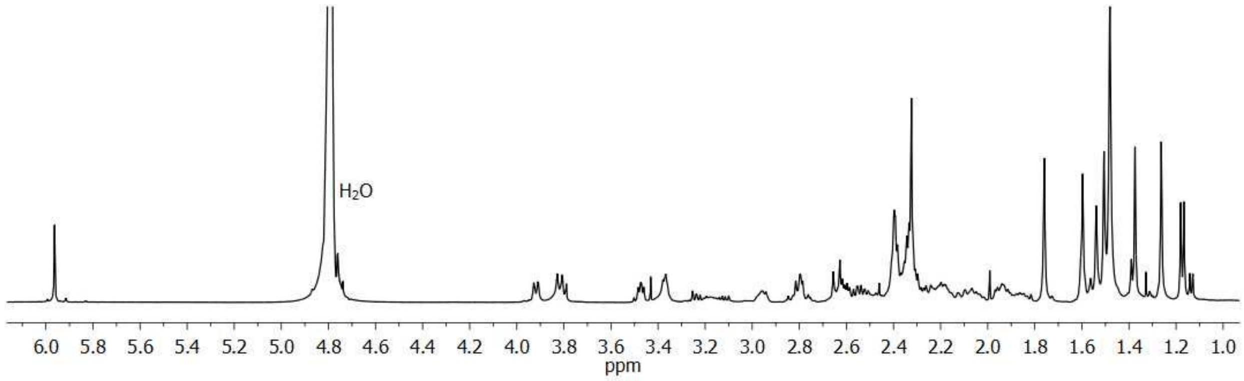

Figure S9 – ^{1}H-NMR of **4** (in D_2O, 270 K, 500 MHz).

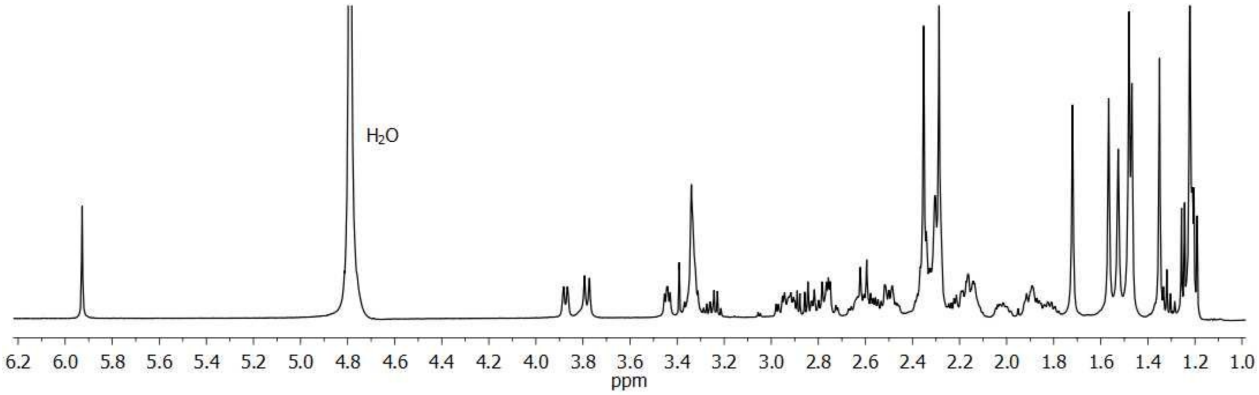

Figure S10 – ^{1}H-NMR of **5** (in D_2O, 270 K, 500.25 MHz).

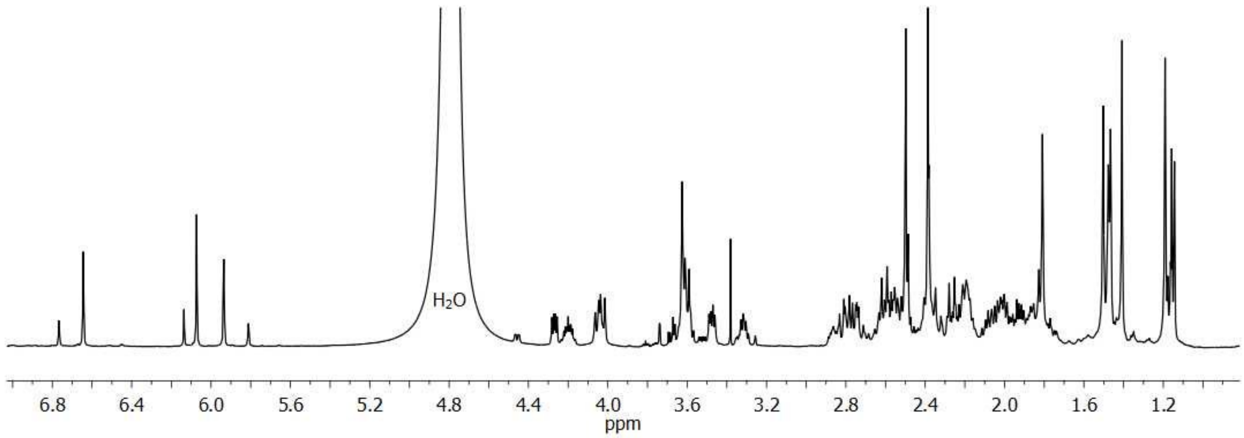

Figure S11 – ^{1}H-NMR of **1-H^{+}** (in D_2O, 270 K, 500.25 MHz).

Table 1. Cartesian coordinates of the QM/M optimized **1-H+**.

N	0.92640000	1.77080000	-1.60940000
C	1.81440000	2.79520000	-1.74800000
C	-0.29220000	2.07460000	-2.23660000
C	-0.28190000	3.60050000	-2.54570000
C	1.27410000	3.84110000	-2.73380000
C	-1.34860000	1.21080000	-2.49340000
C	-1.24900000	-0.22260000	-2.18170000
C	-2.22240000	-1.39050000	-2.59850000
C	-1.44900000	-2.68640000	-2.03980000
H	-2.06610000	-3.01710000	-1.19540000
C	-0.09540000	-2.16670000	-1.46850000
N	-0.21290000	-0.67740000	-1.50100000
C	0.36380000	-2.53490000	-0.00420000
N	1.50230000	-1.56690000	0.25430000
C	2.47560000	-2.06950000	0.98970000
C	1.08500000	-3.96380000	0.27610000
C	2.17650000	-3.55410000	1.37470000
C	3.70510000	-1.34600000	1.33070000
C	3.93930000	-0.03040000	0.91870000
N	3.00380000	0.73000000	0.17610000
C	3.48530000	1.97270000	-0.09430000
C	3.01730000	2.87810000	-1.04650000
H	3.64500000	3.74460000	-1.20630000
H	0.75510000	-2.36510000	-2.12170000
Co	1.32900000	0.13230000	-0.64390000
H	-0.84470000	3.84120000	-3.45280000
C	1.69300000	3.44900000	-4.15750000
H	1.36410000	2.41730000	-4.40880000
H	2.79890000	3.48800000	-4.26290000
H	1.24200000	4.14340000	-4.89870000
C	1.76180000	5.26740000	-2.46320000
H	2.85090000	5.35680000	-2.66290000
H	1.59050000	5.56970000	-1.41010000
H	1.24980000	5.98290000	-3.13920000
C	0.09450000	-5.05910000	0.70540000
H	0.59400000	-6.03480000	0.84740000
H	-0.69190000	-5.18770000	-0.06470000
H	-0.40850000	-4.82810000	1.66300000
C	1.79750000	-3.68290000	2.86920000
H	0.72760000	-3.47250000	3.03230000
H	2.34100000	-2.91860000	3.46650000
H	3.08050000	-4.14690000	1.22770000
C	2.09810000	-5.07050000	3.46720000
H	1.69420000	-5.87240000	2.81920000
H	1.58360000	-5.15860000	4.44810000
C	3.56930000	-5.28910000	3.68880000
O	4.16560000	-6.12550000	3.02520000
N	4.26630000	-4.52250000	4.68160000
H	3.88320000	-4.79860000	5.61380000
H	5.27630000	-4.79470000	4.67790000
C	1.84730000	-4.43870000	-1.00170000
H	2.50640000	-3.62030000	-1.36380000
H	1.13850000	-4.65140000	-1.82170000
C	2.70710000	-5.67210000	-0.82990000
O	2.19600000	-6.78130000	-0.84130000
N	4.13040000	-5.55560000	-0.72590000
H	4.59370000	-4.61980000	-0.73620000
H	4.72250000	-6.40900000	-0.61890000
C	-0.77890000	-2.19780000	0.98270000
H	-1.53470000	-3.00400000	1.04480000
H	-1.33460000	-1.29470000	0.67120000
H	-0.39540000	-1.97220000	1.99380000
C	-1.34670000	-3.92890000	-2.94690000
H	-2.35810000	-4.19770000	-3.31520000
H	-1.04010000	-4.79180000	-2.32470000
C	-0.40850000	-3.86000000	-4.13310000
N	-0.24900000	-5.02490000	-4.95430000
H	0.21790000	-5.75480000	-4.36970000
H	0.40690000	-4.80550000	-5.73920000
O	0.20250000	-2.83750000	-4.40730000
C	-2.45400000	-1.37110000	-4.12160000
H	-1.53160000	-1.07850000	-4.66340000
H	-3.24890000	-0.66110000	-4.41280000
H	-2.79820000	-2.35010000	-4.50420000
C	-2.55640000	1.80560000	-3.18180000
H	-3.41050000	1.12910000	-3.26260000
H	-2.28220000	2.11960000	-4.21070000
H	-2.93180000	2.68390000	-2.61930000
C	5.27670000	0.83420000	0.99370000
C	4.69290000	2.29520000	0.76640000
H	5.38160000	2.96130000	0.24100000
C	6.15840000	0.40720000	-0.21160000
H	5.56290000	0.47290000	-1.14860000
H	6.44140000	-0.65970000	-0.10160000
C	7.43470000	1.19060000	-0.43880000
O	7.74650000	2.12920000	0.27750000
N	8.29310000	0.82590000	-1.52290000
H	8.04580000	0.02900000	-2.15080000
H	9.17680000	1.35370000	-1.69880000
C	6.06070000	0.69810000	2.30860000

H	6.70540000	-0.20300000	2.32160000
H	5.36860000	0.66990000	3.17620000
H	6.74170000	1.55960000	2.45790000
C	4.24480000	3.00270000	2.06740000
C	3.57830000	4.37450000	1.85830000
H	2.65490000	4.27050000	1.24990000
H	3.26360000	4.76640000	2.84900000
C	4.51420000	5.37560000	1.23980000
N	5.74550000	5.70220000	1.89310000
H	6.52270000	5.48690000	1.22790000
H	5.75310000	6.73410000	2.05950000
O	4.21710000	5.92260000	0.18790000
C	4.78480000	-2.07270000	2.10200000
H	5.72850000	-2.08910000	1.52470000
H	4.56440000	-3.12480000	2.32000000
H	4.92870000	-1.59730000	3.09170000
C	-3.59000000	-1.30340000	-1.81000000
H	-3.36620000	-1.28760000	-0.73640000
H	-4.13790000	-0.37470000	-1.98950000
C	-4.57560000	-2.47780000	-2.09370000
H	-4.13950000	-3.43570000	-1.79710000
H	-4.82290000	-2.53290000	-3.16260000
C	-5.85180000	-2.30310000	-1.27380000
O	-5.96950000	-2.77550000	-0.10520000
N	-6.85300000	-1.57360000	-1.86710000
H	-6.70280000	-1.21330000	-2.80190000
C	-8.16470000	-1.33850000	-1.23380000
H	-8.46140000	-2.25710000	-0.71120000
H	-8.89470000	-1.15520000	-2.02950000
C	-8.19980000	-0.15020000	-0.24640000
N	-7.32220000	-0.45850000	0.90210000
C	-6.84940000	0.51250000	1.73390000
O	-7.11500000	1.74130000	1.60440000
C	-6.01400000	-0.01120000	2.92250000
H	-5.60720000	-0.99770000	2.67700000
N	-4.90620000	0.91810000	3.24500000
C	-3.82720000	1.00320000	2.42120000
O	-3.71690000	0.29550000	1.37810000
N	-0.28120000	1.31770000	3.04530000
C	0.71870000	0.85940000	2.22440000
H	1.68380000	0.53200000	2.57050000
N	0.29670000	0.86820000	0.95150000
C	-1.01050000	1.36250000	0.96020000
C	-0.91810000	4.33660000	-1.34420000
H	-0.17890000	4.47110000	-0.52560000
H	-1.72960000	3.71130000	-0.91450000
C	-1.54640000	5.68230000	-1.73240000
H	-2.38610000	5.51240000	-2.44040000
C	-2.07380000	6.40140000	-0.52080000
O	-1.55700000	7.45220000	-0.16830000
N	-3.20550000	5.87980000	0.19160000
H	-3.50110000	6.57280000	0.91730000
H	-2.88710000	5.02980000	0.70890000
C	2.37770000	-0.47630000	-2.09490000
N	3.05260000	-0.85030000	-2.99740000
H	3.53030000	2.35130000	2.61670000
H	5.11940000	3.15880000	2.73260000
H	-6.92910000	-1.40160000	0.94620000
H	-7.80800000	0.75150000	-0.73770000
C	-5.19800000	1.76810000	4.43250000
H	-5.08220000	2.83080000	4.19310000
H	-4.51880000	1.51430000	5.26000000
C	-6.84870000	-0.01480000	4.23140000
H	-6.43530000	-0.75730000	4.92550000
H	-7.89810000	-0.26590000	4.05130000
C	-6.66340000	1.42000000	4.78370000
H	-7.33660000	2.10710000	4.26360000
H	-6.85170000	1.48630000	5.85980000
C	-9.64660000	0.13270000	0.21220000
H	-10.09010000	-0.75380000	0.68370000
H	-10.27520000	0.42710000	-0.63850000
H	-9.65020000	0.95350000	0.93560000
H	-0.21880000	1.40930000	4.05130000
C	-2.73170000	2.02790000	2.81450000
H	-2.67840000	2.13870000	3.90370000
H	-3.04760000	3.00310000	2.41820000
C	-1.39630000	1.63760000	2.25760000
H	-1.61260000	1.41900000	0.07430000
H	-0.80230000	6.32480000	-2.24280000

References

[S1] O. M. G. Müller, *Naturforsch.* **1966**, 1159.
[S2] C. Männel-Croisé, F. Zelder, *Chem. Commun.* **2011**, *47*, 11249.
[S3] B. P. Gangamani, V. A. Kumar, K. N. Ganesh, *Tetrahedron* **1996**, *52*, 15017.
[S4] S. Jordan, C. Schwemler, W. Kosch, A. Kretschmer, E. Schwenner, U. Stropp, B. Mielke, *Bioorg. Med. Chem. Lett.* **1997**, *7*, 681.
[S5] K. Zhou, F. Zelder, *Angew. Chem. Int. Edit.* 2010, 49, 5178-5180**.**
[S6] M. J. Frisch, G. W. Trucks, H. B. Schlegel, G. E. Scuseria, M. A. Robb, J. R. Cheeseman, G. Scalmani, V. Barone, B. Mennucci, G. A. Petersson, et al., Gaussian, Inc., Wallingford, CT, USA, **2010**.
[S7] S. Dapprich, I. Komáromi, K. S. Byun, K. Morokuma, M. J. Frisch, *J. Mol. Struct.: THEOCHEM* **1999**, *461*, 1-21.
[S8] T. Vreven, K. Morokuma, Ö. Farkas, H. B. Schlegel, M. J. Frisch, *J. Comput. Chem.* **2003**, *24*, 760-769.
[S9] T. Vreven, K. S. Byun, I. Komáromi, S. Dapprich, J. A. Montgomery, K. Morokuma, M. J. Frisch, *J. Chem. Theory Comput.* **2006**, *2*, 815-826.
[S10] T. V. F. Clemente, M. J. Frisch, in Quantum Biochemistry, C. Matta Wiley VCH, Weinheim, **2010**, pp. 61-84.
[S11] A. D. Becke, *J. Chem. Phys.* **1993**, *98*, 5648-5652.
[S12] C. Lee, W. Yang, R. G. Parr, *Phys. Rev. B* **1988**, *37*, 785.
[S13] T. H. Dunning, P. J. Hay, H. F. Schaefer, in Modern Theoretical Chemistry, Vol. 3, Plenum Press New York, **1977**, pp. 1-28.
[S14] P. J. Hay, W. R. Wadt, *J. Chem. Phys.* **1985**, *82*, 270-283.
[S15] P. J. Hay, W. R. Wadt, *J. Chem. Phys.* **1985**, *82*, 299-310.
[S16] W. R. Wadt, P. J. Hay, *J. Chem. Phys.* **1985**, *82*, 284-298.
[S17] A. K. Rappé, C. J. Casewit, K. S. Colwell, W. A. Goddard III, W. M. Skiff, *J. Am. Chem. Soc.* **1992**, *114*, 10024-10035.
